# The impact of NHS charging regulations on healthcare access and utilisation among migrants in England: a systematic review

**DOI:** 10.1186/s12889-023-15230-9

**Published:** 2023-02-28

**Authors:** Nazanin Rassa, Margaret McCarthy, Seb Casalotti, Claire Zhang, Fatima Wurie, Colin Brown, Ines Campos-Matos

**Affiliations:** 1 NR. Foundation Year 2 Doctor, Southmead Hospital, North Bristol NHS Trust, Bristol, UK; 2 Board On Health Sciences Policy, The National Academies of Sciences, Engineering, and Medicine, 500 5Th St NW, Washington, D.C. 20001 USA; 3 SC. Foundation Year 1 Doctor, Queen’s Hospital, Barking Havering and Redbridge University Trust, Romford, UK; 4 CZ. Programme Manager - Population Health Metrics (Inclusion Health), Office for Health Improvement and Disparities, Department of Health and Social Care, 39 Victoria Street, London, SW1H 0EU UK; 5 FW. National Migrant Health Lead & Inclusion Health Officer, Office for Health Improvement and Disparities, Department of Health and Social Care, 39 Victoria Street, London, SW1H 0EU UK; 6 CB. Director of Clinical and Emerging Infections (Interim) & Deputy Director of Clinical & Public Health Group - HCAI, Fungal, AMR, AMU, & Sepsis Division (Interim) Director of WHO Collaborating Centre for Reference & Research On Antimicrobial Resistance and Healthcare Associated Infections, UK Health Security Agency, London, UK; 7 IC. Deputy Director for Inclusion Health, Addictions and Inclusion Directorate, Office for Health Improvement and Disparities, Department of Health and Social Care, National COVID-19 Epidemiology Team, 39 Victoria Street, London, SW1H 0EU UK

**Keywords:** Policy, Healthcare access, Migrant health, Cost recovery

## Abstract

**Background:**

The NHS Charges to Overseas Visitors Regulations 2015 outline when healthcare costs should be recuperated from overseas visitors in England. National and global stakeholders have expressed concerns that charging may exacerbate health inequalities and undermine public health efforts especially among vulnerable migrant groups. This review aims to systematically describe the evidence regarding the impact of NHS charging regulations on healthcare access and utilisation and health outcomes for migrants in England.

**Methods:**

A systematic search of scientific databases and grey literature sources was performed. Quantitative and qualitative studies, case studies and grey literature published between 1 January 2014 and 1 April 2021 were included. Screening, data extraction and quality appraisal were carried out in accordance with PRISMA guidelines.

**Results:**

From the 1,459 identified studies, 10 were selected for inclusion. 6 were qualitative, 3 were mixed methods and 1 was quantitative. The evidence is lacking but suggests that fears of charging and data sharing can deter some migrants from accessing healthcare. There is also evidence to suggest a lack of knowledge of the charging regulations among patients and healthcare professionals is contributing to this deterrence.

**Conclusions:**

Further independent research supported by strengthening of data collection is required to better understand the effects of charging on healthcare and health outcomes among vulnerable migrants. Our findings support improved training and communication about NHS Charging Regulations for patients and professionals.

**Supplementary Information:**

The online version contains supplementary material available at 10.1186/s12889-023-15230-9.

## Background

It is estimated that nearly one seventh of the world’s population lives in a location that is different to where they were born [1]. In the United Kingdom (UK), the proportion of people born outside of the country has increased from 7.5 million to 10 million within the last 10 years [2]. Many of the countries receiving high numbers of international migrants have recognised the right to universal healthcare through their national constitutions [3] and international instruments such as the United Nations Sustainable Development Goals [4].

In recent years there has been increased international attention paid to global migration. This discourse has often centered the balance between upholding international commitments to universal healthcare while managing demands on national public services [1]. In the context of the UK’s National Health Service (NHS) this commitment is underlined through the NHS constitutional principle that access to healthcare is based on clinical need and that “NHS services are free of charge, except in limited circumstances sanctioned by Parliament” [5].

These circumstances are outlined in the NHS Charges to Overseas Visitors Regulations 2015, hereon referred to as ‘NHS Charging Regulations’ which outline the circumstances when healthcare services are chargeable. Under these regulations, NHS trusts and other healthcare providers are responsible for recovering costs from overseas visitors at 150% of the service tariff for most secondary and tertiary care services [6]. Charging exemptions are in place for specific groups of individuals as well as “urgent and immediately necessary” treatment such as emergency or maternity care (Appendix A) [7].

NHS Charging Regulations mainly focus on identifying chargeable patients and recuperating healthcare costs from overseas visitors [8, 9]. Data collected on overseas visitor debts have also been used for immigration enforcement purposes [6].

Organisations such as the United Nations, the Lancet and the British Medical Association have expressed concerns that charging practices may exacerbate health inequalities and undermine public health effortsespecially among vulnerable migrant groups [10, 11]. A number of Royal Colleges have called for the suspension of NHS charging regulations pending a full independent evaluation [12–16].

A government review of amendments made to NHS Charging Regulations in 2017, with a focus on impacts on vulnerable groups, reported that there was no evidence of deterrent effect [17]. The findings of this review have not been released to the public [18]. At present, empirical research into the effects of NHS charging regulations is lacking. This review aims to systematically describe the evidence and highlight gaps in research regarding the impact of NHS charging regulations on healthcare access and utilisation for migrants in England.

## Methods

The study was conducted in line with the Preferred Reporting Items for Systematic Reviews and Meta-Analyses (PRISMA) guidelines (Appendix B).

### Search strategy

The systematic strategy was outlined in PROSPERO protocol CRD42021256015.

Searches were conducted in EBSCO CINAHL, EBSCO Health Business Elite, Ovid Embase, Ovid Emcare, Ovid HMIC, Ovid Medline, Social Policy and Practice, Web of Science. A concurrent grey literature search was carried out using National Institute for Health and Care Excellence (NICE), National Institute for Health Research (NIHR) Journal Library, Google, Trip database, Nesta, Nuffield Trust, King’s Fund and Health Foundation. The reference lists of seminal studies were also searched manually.

An example of a full search strategy is presented in Appendix B. Searches were performed between April and May 2021.

### Study selection

Title and abstract screening were carried out by authors NR and MM. Where discrepancies were found, consensus was reached with a third author, SC. Full text screening was performed in the same manner.

Eligible study types included quantitative or qualitative studies, case studies and grey literature which were conducted in English and published between 1 January 2014 and 1 April 2021. The start date was chosen to capture any evidence published in anticipation of the implementation of the Immigration Act in July 2014 which marked the start of NHS Charging Regulations.

The intervention of interest was notification of charge, receipt of invoices, or payments made for healthcare as part of the NHS Charging Regulations.

Studies were included if they measured healthcare access (e.g., barriers directly or indirectly due to charging regulations) or utilisation (e.g., number or length of presentations, appointments or admissions) across any NHS service. Secondary outcomes of interest were measures of morbidity and mortality (e.g., rate of disease occurrence or progression, death or disability), likely affected by healthcare utilisation.

Studies were excluded if they did not include at least one refugee, asylum-seeker, refused asylum seeker, undocumented migrant, or person not ordinarily resident of any age living in England. Studies that included perspectives from healthcare professionals who currently or previously worked with the target population in a healthcare setting were also included.

### Data extraction

A data extraction form was designed according to the Population, Intervention, Comparator, Outcomes, Study design (PICOS) framework. This was piloted on three studies before revision and application to all studies through independent extraction by NR and MM. Data was extracted on study design, aim, migrant subgroup, healthcare setting, nature of charge (timing, scale, method of billing, how and whether it was paid), effects on healthcare access and utilisation, and morbidity or mortality effects.

### Quality appraisal

Qualitative studies were appraised using the NICE quality appraisal checklists H [19–22] and quantitative studies using checklist G [23, 24]. Mixed methods studies were appraised using both checklists (Appendix C).

### Data analysis

Codes and themes for data analysis were developed through discussion between authors NR and MM. Findings are presented as a narrative synthesis.

## Results

### Study selection

**Fig. 1:**
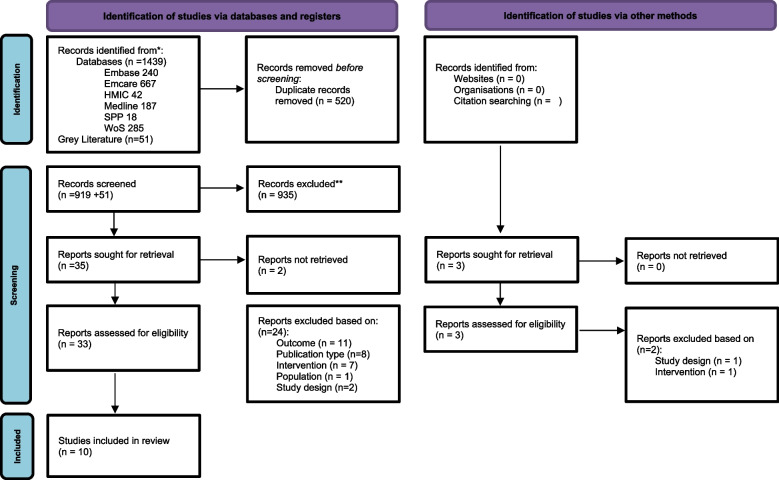
PRISMA flow diagram [25]

From the 1,459 identified studies, 10 were selected for inclusion after screening as illustrated in Fig. 1.

### Study characteristics

Study characteristics are presented in Table 1. Among the 10 included studies, 6 were qualitative [26–31], 3 mixed-methods [32–34] and one quantitative [35]. Six studies were conducted in London [27, 29–31, 34, 35], three in the United Kingdom [26, 28, 32] and one in England and Wales [33]. Studies were published between 2015 and 2021.

**Table 1:** Characteristics of included studies

Study type	Reference	Location	Population	Methods	Setting	Scale of charge (£)	Timing of payment	Method of presenting charge	Information about repayment plans or challenging charges	Outcomes
Qualitative	(Maternity Action, 2018) [30]	London/ Nearby	Migrant women – undocumented migrants, EU citizen, refused asylum seekers, those with temporary visas	Interviews	Maternity care	£2100-£6900	Mostly billed after pregnancy but some received bills in advance of receiving treatment One-off charges 1–30 days to pay	Letters, invoices, and telephone calls	Information offered to some patients but not all	Perceived incorrect charging Requiring external assistance Inability to pay Refusal care Deterrence Fear of further charging Fear of data sharing Mental health impact
Qualitative	(Feldman, 2020) [27]	London/ Nearby	Migrant women	Semi-structured interviews	Maternity Care	£2000 to £6500	Mostly billed after pregnancy (up to a year) but some during pregnancy One-off charges	Letters, invoices and telephone calls	Information offered to some patients but not all	Deterrence Fear of further charging Fear of data sharing Inability to pay Perceived Incorrect charging Refusal of care Requiring external assistance Mental health impact
Qualitative	(Gardner, 2021) [28]	UK	Migrants including refugees, undocumented migrants, temporary visa holders and those with indefinite leave to remain	Online survey	Not described	Not described	Not described	Not described	Not described	Fear of charging Fear of data sharing
Qualitative	(Healthwatch Hackney, 2020) [29]	Hackney, London	Migrant women including refugees	Semi-structured interviews	Maternity care	£1000 to £15,000	Not detailed for all charges One case of a cumulative charge for two pregnancies as a one-off charge retrospectively	Not described	Not described	Fear of charging Fear of data sharing Deterrence
Mixed-Method	(Murphy et al., 2020) [32]	United Kingdom (mostly London)	Children’s healthcare practitioners (HCPs)	Digital survey	Pediatric Care	Not described	Not described	Not described	Not described	Deterrence Fear of data sharing Refusal of care HCP beliefs and confidence navigating regulations Physical health impact Perceived incorrect charging
Qualitative	(Nellums et al., 2021) [31]	East London	Migrant women – all undocumented	Semi-structured interviews	Maternity	£3072 to £11,500	Not described	Not described	Not described	Delay in healthcare access Fear of charging Fear of data sharing Inability to pay Lack of information
Quantitative	(Potter et al., 2020) [35]	Barts Health NHS Trust, London	Adults born outside of the UK on the LTBR	Infectious disease surveillance data	Secondary care (TB treatment)	Not described	Not described	Not described	Not described	Delay in diagnosis and treatment
Qualitative	(Doctors of the World, 2020) [26]	United Kingdom	Migrant women – asylum seeker, refused asylum seeker, other	Interviews	Secondary care (Cancer services)	One case of £8,397	One off charge in one case study Upfront payment before treatment given in one case study	Invoice	Monthly repayments in one case study	Delay in treatment Refusal of care Perceived incorrect charging Deterrence
Mixed-methods	(Shortall, 2015) [34]	London	Pregnant migrant women	Telephone questionnaire	Maternity care	£1500-£6000 One-off charges	Retrospective	Not described	One case of a patient paying monthly	Delay in clinic attendance Fear of further charging Fear of data sharing
Mixed-methods	(Patients not Passports, 2020) [33]	England and Wales	Members of advocacy organisations	Online survey followed by semi-structured telephone interviews	Not described	Not described	Not described	Not described	Not described	Deterrence Fear of charging Fear of data sharing Refusal of care Lack of awareness of exemptions

Six studies covered migrants who had experienced charging or were expecting to be charged [26–31]. One study used a telephone questionnaire [34] and another a digital survey [32]. Another study analysed infectious disease surveillance data [35].

Half of studies described charging in the context of maternity care [27, 29–31, 34]. Remaining studies described findings in other secondary care settings [26, 35]. Four studies sampled migrant women [27, 26, 30, 34]while one focused on undocumented migrant women [31]. Only two studies presented data on immigration status [28, 30].

Two studies included views of healthcare professionals [28] and members of migrant support organisations [29, 33].

Almost all studies were qualitative. Only one study presented quantitative metrics of access and utilisation. This was an ecological study which measured delay to tuberculosis diagnosis by comparing the time between symptom onset and treatment initiation before and after implementation of charging practices [35]. The three mixed methods studies presented quantitative data only for findings not relating to healthcare access or utilisation [32–34].

### Nature of charging

Details of charging practices were only described in the context of maternity care. Costs for maternity ranged between £1,000—£11,500 [27, 29, 30]. Two studies suggested that there was considerable variation in the charges for standard maternity care. However, it was not clear how the level of care patients received was evaluated [29, 30].

Most individuals were charged after receiving healthcare but some experienced charging before or during access to services [26, 30]. The longest period between accessing care and receiving an invoice was one year [27, 30].

Most charges were communicated by post, however some patients also received phone calls and emails. Some patients received a single invoice whereas others received multiple reminders [26, 27, 30]. The contents of these invoices were variable. They were often not itemised by service provided [27, 29] and some included charges for care related to past pregnancies or other episodes of care [30]. Some patients were offered information about repayment plans but this was inconsistently reported [26, 30, 34]. No studies reported whether patients were given information about how to challenge instances of perceived incorrect charging.

We did not identify any studies that used comparator groups, such as a non-charging comparator policy nor any studies comparing the effects of the NHS Charging Regulations in England to the implementation of similar regulations in other UK devolved administrations.

### Effects on healthcare access and utilisation

#### Fear of data sharing

Migrants self-reported fears that their unpaid debts would lead to data sharing for immigration enforcement purposes [28, 29, 33], negatively affect their immigration applications [26] or result in deportation [27, 32]. Some migrants felt intimidated by letters or phone calls from debt collection agencies suggesting that they would be reported to the Home Office if they failed to pay [27, 30]. One study added that fear was more evident among undocumented migrants [28].

#### Fear of further charging

Fear of further charging was observed in migrants who were informed about charges for the first time as well as those who had experienced charging in the past [27, 28, 30, 34, 33]. Fears persisted among migrants who were exempt from charging [24] even when they were aware of the charging exemptions that applied to them [33].

#### Delay and deterrence

Seven studies described the deterrent effects of NHS charging regulations [26, 27, 29, 30, 32, 33]. Some migrants perceived a need for healthcare but chose to delay seeking care or turned to home remedies [29].

Late presentation to antenatal care was explored in four studies. This was measured in samples of 20 and 35 individuals as self-reported delay or first antenatal appointment later than the recommended national target [31, 34]. Three case studies included individuals who decided not to access any antenatal care after learning of charging practices [27, 30].

Two studies measured perceived late attendance assessed by healthcare professionals [32] or individuals working with migrant support organisations [33] with sample sizes of 200 and 70 respectively.

In an ecological analysis, tuberculosis care median time to treatment was found to increase from 69 to 89 days among non-UK born patients following the introduction of NHS charging regulations [35]. Delay was also described by individuals working for migrant support organisations in the context of diabetic and cancer care [33]. One paper reported an average delay of 36 weeks in care deemed by the authors to be urgent or immediately necessary, however details of how this was measured were no reported [26].

### Inability to pay

Four studies found that most migrants who experienced charging for antenatal care reported inability to pay [29–31, 34].

One study found that 10 out 16 women who were interviewed could not pay their healthcare charges. Contributing factors included barriers to obtaining financial advice, lack of income [30] and destitution [34, 30, 31]. In cases where repayment plans were established for antenatal care, often patients could not afford the monthly payments [30]. This study also highlighted the relationship between inability to pay and precarious immigration status as ability to pay was only observed in two patients who had spousal visas.

#### Perceptions of incorrect charging among stakeholders

Studies highlighted concerns from healthcare professionals and individuals working for migrant support organisations about perceived incorrect charging of exempt patients [26, 32]. One study by Doctors of the World found that 6 out of the 27 patients whom they assessed as being exempt were charged for healthcare costs [26].

Two studies attributed this partly due to barriers in accessing identification or changes to immigration status since last assessment [30, 32]. One study highlighted how administrative errors or changes to immigration status can also contribute to perceived incorrect charging. In some cases, healthcare charges were not reassessed or cancelled when records were updated. Some patients also reported being charged for past treatments despite having been eligible for free healthcare at the time of accessing the service [30].

#### Care denied by provider

Two studies found cases of individuals being asked to pay before they could access healthcare [26, 30]. In one case, an individual reported she was informed that her future antenatal care would be cancelled if she was unable to pay [30].

The other study highlighted two cases of care denied to patients because of inability to pay in the context of new cancer diagnoses. In one case a patient received urgent secondary care treatment but was discharged from hospital once his immigration status was confirmed and he had been assessed as unable to pay. This patient was discharged without community referrals despite being homeless and having a new diagnosis of terminal cancer. In another case, a patient with a diagnosis of nodal malignant melanoma had her tertiary care appointments for immunotherapy cancelled due to inability to pay due to an absence of income [26].

#### Lack of information and knowledge

Patients are often unaware that they are liable for or exempt from healthcare charging [30, 31, 33]. In the context of maternity care, patients reported that they were often not informed to expect charging prior to or after giving birth [27]. In one study where 16 women were interviewed, none of the participants could recall being provided with signposting to information to help them navigate charges [30]. This lack of information was sometimes compounded by a lack of translation services [29].

Patients often required assistance from charities or other external actors in order to obtain advice around challenging charges and establishing repayment plans [26, 30].

#### Lack of confidence in applying charges

Healthcare professionals highlighted a lack of confidence and knowledge when navigating NHS Charging Regulations compounded by an absence of formal training [29, 32].

One study found that healthcare professionals expressed concerns about the potentially adverse health effects of NHS Charging Regulations and that some disagreed with the expectation that they should have a role in implementing charging. They also highlighted frustrations that administrative staff would sometimes act independently, without informing clinicians and present charges to unwell patients worsening distress and fear [32].

#### Effects on morbidity and mortality

Three studies included effects of charging on morbidity and mortality.

One study documented 12 cases of health complications perceived by healthcare professionals to stem from deterrence and delays to accessing care. This included two cases of intrauterine death and four cases of children presenting in a critical condition [32]. Two studies described self-reported anxiety and low mood among migrants experiencing charging [26, 30].

### Quality of evidence

As outlined in Appendix C, seven studies were of low quality [26–30, 33, 34]. Few studies adequately detailed their analysis or the limitations of their research.

Seven studies were from authors or organisations affiliated with migrant advocacy work and participants were often recruited by sampling migrants accessing specific support services but these biases were mostly unexplored [26, 28–30, 33, 34] and rarely addressed when recognised [26]. Some of the studies also used anonymised vignettes but did not detail how these were selected [27, 29, 30, 33].

While qualitative evidence allowed for a richer exploration of the effects of charging in multiple facets of healthcare access, the details of charging as the main intervention were not always clear.

## Discussion

### Summary of findings

This review found that existing analyses regarding the impact of charging on migrant health are of poor quality and predominantly carried out for advocacy purposes. Further independent research is required to better understand and quantify the effects of charging on healthcare and health outcomes among vulnerable migrants. However, the existing evidence suggests that charging deters some migrants from accessing some healthcare services, sometimes leading to delays in diagnosis and treatment even when healthcare is deemed urgent or immediately necessary.

Additionally, migrant support organisations perceived their service users to have been charged incorrectly in situations where exemptions applied. This was sometimes attributed to difficulties in determining immigration status. There is strong evidence to suggest that patients and healthcare professionals lacked knowledge and confidence in navigating NHS Charging Regulations. Some healthcare professionals also raised concerns about the negative effects of charging practices on patients as well as conflicts with professional values.

### Existing literature

Our findings are consistent with reports produced by grassroots organisations which have highlighted the potentially negative effects of the NHS Charging Regulations [1, 11, 12, 36, 37]. The fear resulting from real or perceived links between healthcare systems and immigration enforcement have been widely documented to deter and reduce access to healthcare among some migrant groups both within the UK [1, 38] and other European countries [3].

A report on the access to healthcare for undocumented migrants in 11 European countries by the Platform for International Cooperation on Undocumented Migrants presents similar findings to this review. The main findings include fears of paying unaffordable healthcare costs, lack of information among patients, healthcare providers and administrators; application of more restrictive versions of healthcare policies which deviate from national guidance and deterrence and delay in access due to strict administrative requirements of identification or proof of address documents [3].

Previous national reports have also highlighted that migrants are denied care by providers even in circumstances of urgent or immediately necessary treatment contrary to guidance [36, 39]. In the context of maternity care, it has been suggested in one confidential enquiry that fear and deterrence resulting from NHS Charging Regulations may contribute to patient mortality [40]. However, at present little empirical research exists quantifying the effects of charging practices on patient morbidity or mortality.

Lack of knowledge about NHS charging regulations among patients and healthcare professionals resonate with a national report from the non-profit organisation Medact finding that 67% of 99 NHS trusts analysed provided no training to staff about the regulations [41].

Furthermore, stakeholders have previously highlighted that NHS Charging Regulations may contradict professional values and duties [1, 42] especially when pressure from NHS Trusts interfere with clinical judgements [11], despite guidance that implementation of the regulations should not delay urgent or immediately necessary treatment.

### Implications for further research

The majority of existing evidence consists of reports from migrant advocacy organisations which primarily aim to inform advocacy work. These are small-scale studies which are context-specific and difficult to generalise to England more broadly. In order to address this, further independent academic research is required.

Analyses using routine sources of data such as electronic health records [43] or large-scale cohort surveys could improve the current gap in quantitative evidence. Hospital episode statistics, a form of patient level data, are already widely used to inform healthcare research [44]. Though the challenge of using such data while also maintaining security and patient confidentiality has been internationally recognised, the OECD maintains that even sensitive patient data can be used for research and monitoring as it is integral to improving healthcare outcomes and systems [45].

Studies in England have already used such data to quantify healthcare utilisation for migrants [46], including specifically for refugees [47]. However, in the context of charging routine data collection would need to be extended to identify all patients potentially affected by NHS Charging regulations. This would be challenging due to fear of data sharing for immigration enforcement [48].

Further research into the effects of charging on morbidity and mortality, as well as which migrant groups are more impacted, would aid a more comprehensive understanding of the effects of NHS Charging Regulations.

### Implications for policy and practice

Where providers of relevant services work to identify chargeable patients, it is imperative to use consistent methods of assessment. These efforts should be dynamic in order to recognise any changes to a patient's status which may affect their eligibility for free NHS healthcare. Delivery of comprehensive and accessible information to and to healthcare professionals could be improved by partnering with local community organisations within a multiagency approach.

Strengthening ongoing policy evaluation using systematic data collection across NHS Trusts could improve understanding of how providers of relevant services identify chargeable patients and apply NHS Charging Regulations. This could potentially highlight areas for improvement, address mistrust among patients and clarify regulations for healthcare professionals.

Wider considerations include alternate ways to supplement charging, including means-testing, exemptions, and considerations of alternative charging models, which has been discussed in a recent Institute for Public Policy Research publication [37]. Lessons can also be drawn from other European countries such as Italy’s legislation which prevents confidential patient data being used for immigration enforcement or the use of wider understandings of “urgent” medical treatment such as in Belgium where this encompasses preventative treatment of illness which could cause harm for the patient or their social circle [3].

### Strengths and limitations

To our knowledge, this is the first study to systematically review evidence of the effects of NHS Charging Regulations on healthcare access and utilisation among migrants in England. The quality of evidence was poor and the nature and effects of charging have been mostly studied in the context of maternity care, which limited our ability to make robust conclusions. Additionally, no included studies disaggregated their findings by immigration status, so we adapted our PROSPERO protocol and expanded our population of focus to all migrants.

To retain as many studies as possible we intended to extract data relating to England in UK-wide studies. However, results were not disaggregated by devolved administration in studies that met inclusion. Therefore, we extracted and synthesised all findings relating to the effects of charging practices across the UK. Findings should therefore be interpreted with caution as charging practices in Wales, Scotland and Northern Ireland are governed by separate legislation under their devolved administrations [7].

## Conclusions

This systematic review has identified a limited body of low-quality evidence, which limited our ability draw firm conclusions about the impact of NHS charging regulations on migrants’ healthcare access and utilisation in England. We found evidence of fear and deterrence among some migrants accessing NHS healthcare despite the existence of exemptions and provisions in the charging regulations to prevent this. This was compounded by inability to pay, perceived inconsistencies in application of charging and a lack of knowledge about how to navigate these policies among patients and healthcare professionals. Few studies highlighted adverse effects of charging on morbidity and mortality.

Additional evaluation of training and public communication could improve outcomes for patients and healthcare professionals by continuing to improve knowledge and understanding of NHS Charging Regulations. Further independent research supported by improvements to data collection is required to understand the effects of charging on healthcare, morbidity and mortality across the NHS. Finally, given that many of our findings have also been noted in the European context, future policy should consider lessons from other countries that have implemented policies to address similar issues and meet the unmet needs of vulnerable migrant populations.

## Supplementary Information

**Additional file 1: Appendix A.** Exemptions to NHS Charging Regulations. **Appendix B.** Example search strategy. **Appendix C.** Quality appraisal. **Appendix D**. Prisma Checklist.

## Data Availability

The data used in this systematic review can be found through the databases described in the methods of this review. The data sets and analysis are available from the corresponding author on request.

## Abbreviations

NHS: National Health Service
NICE: National Institute for Clinical Excellence
NIHR: National Institute for Health Research
PICOS: Population Intervention Control Outcome Study
PRISMA: Preferred Reporting Items for Systematic Reviews and Meta-Analyses
UK: United Kingdom
