# Supplementary material for: The impact of NHS charging regulations on healthcare access and utilisation among migrants in England: a systematic review
Source: BMC Public Health. 2023 Feb 28;23:403. doi: 10.1186/s12889-023-15230-9 (PMC9971664; doi:10.1186/s12889-023-15230-9)
Supplement: Supplementary file 1 — Additional file 1: Appendix A. Exemptions to NHS Charging Regulations. Appendix B. Example search strategy. Appendix C. Quality appraisal. Appendix D. Prisma Checklist. [file 12889_2023_15230_MOESM1_ESM.docx]

**Appendix A. Exemptions to NHS Charging Regulations**

NHS guidance on exemptions for specific groups of visitors available at <https://www.nhs.uk/nhs-services/visiting-or-moving-to-england/visitors-who-do-not-need-to-pay-for-nhs-treatment/> (Accessed x date).

Department of Health and Social Care, Guidance on implementing the overseas visitor charging regulations available at <https://assets.publishing.service.gov.uk/government/uploads/system/uploads/attachment_data/file/1029984/guidance-on-implementing-the-overseas-visitor-charging-regulations.pdf> (Accessed x date).

**Appendix B. Example search strategy**

Data base: Embase

Search Strategy:

#

1 exp *migrant/

2 exp *migration/

3 (immigrant* or migrant* or "asylum seeker*" or refugee* or "seeking asylum").tw,kw.

4 ("non UK" or "non British" or foreign or "non EEA").tw,kw.

5 ((outside or non) adj1 "European Economic Area").tw,kw.

6 (overseas adj1 (resident* or visitor*)).tw,kw.

7 ((trafficking or trafficked) adj1 (victim* or people)).tw,kw.

8 or/1-7

9 exp *"health care cost"/

10 (charg* or pay* or paid or cost* or afford* or unaffordable or fee#).tw,kw.

11 (health* adj3 bill*).tw,kw.

12 10 or 11

13 *health care access/

14 *health care utilization/

15 (access* adj3 (healthcare or service* or treatment*)).tw,kw.

16 (health* adj2 (utili?ation or utili?ing)).tw,kw.

17 *help seeking behavior/

18 ((help or health* or care) adj seek*).tw,kw.

19 ((barrier* or prevent* or deter or deterred or deterrent) adj5 access*).tw,kw.

20 (NHS or "National Health Service").tw,kw.

21 health disparity/

22 (equit* or inequit* or inequalit* or disparit* or equality).tw,kw.

23 or/13-22

24 emergency care/

25 exp primary health care/

26 secondary health care/

27 tertiary health care/

28 ((primary or secondary or tertiary or emergency) adj care).tw,kw.

29 (GP* or "general practi*").tw,kw.

30 or/24-29

31 United Kingdom/

32 (national health service* or nhs*).ti,ab,in,ad.

33 (english not ((published or publication* or translat* or written or language* or speak* or literature or citation*) adj5 english)).ti,ab.

34 (gb or "g.b." or britain* or (british* not "british columbia") or uk or "u.k." or united kingdom* or (england* not "new england") or northern ireland* or northern irish* or scotland* or scottish* or ((wales or "south wales") not "new south wales") or welsh*).ti,ab,jw,in,ad.

35 (bath or "bath's" or ((birmingham not alabama*) or ("birmingham's" not alabama*) or bradford or "bradford's" or brighton or "brighton's" or bristol or "bristol's" or carlisle* or "carlisle's" or (cambridge not (massachusetts* or boston* or harvard*)) or ("cambridge's" not (massachusetts* or boston* or harvard*)) or (canterbury not zealand*) or ("canterbury's" not zealand*) or chelmsford or "chelmsford's" or chester or "chester's" or chichester or "chichester's" or coventry or "coventry's" or derby or "derby's" or (durham not (carolina* or nc)) or ("durham's" not (carolina* or nc)) or ely or "ely's" or exeter or "exeter's" or gloucester or "gloucester's" or hereford or "hereford's" or hull or "hull's" or lancaster or "lancaster's" or leeds* or leicester or "leicester's" or (lincoln not nebraska*) or ("lincoln's" not nebraska*) or (liverpool not (new south wales* or nsw)) or ("liverpool's" not (new south wales* or nsw)) or ((london not (ontario* or ont or toronto*)) or ("london's" not (ontario* or ont or toronto*)) or manchester or "manchester's" or (newcastle not (new south wales* or nsw)) or ("newcastle's" not (new south wales* or nsw)) or norwich or "norwich's" or nottingham or "nottingham's" or oxford or "oxford's" or peterborough or "peterborough's" or plymouth or "plymouth's" or portsmouth or "portsmouth's" or preston or "preston's" or ripon or "ripon's" or salford or "salford's" or salisbury or "salisbury's" or sheffield or "sheffield's" or southampton or "southampton's" or st albans or stoke or "stoke's" or sunderland or "sunderland's" or truro or "truro's" or wakefield or "wakefield's" or wells or westminster or "westminster's" or winchester or "winchester's" or wolverhampton or "wolverhampton's" or (worcester not (massachusetts* or boston* or harvard*)) or ("worcester's" not (massachusetts* or boston* or harvard*)) or (york not ("new york*" or ny or ontario* or ont or toronto*)) or ("york's" not ("new york*" or ny or ontario* or ont or toronto*))))).ti,ab,in,ad.

36 (aberdeen or "aberdeen's" or dundee or "dundee's" or edinburgh or "edinburgh's" or glasgow or "glasgow's" or inverness or (perth not australia*) or ("perth's" not australia*) or stirling or "stirling's").ti,ab,in,ad.

37 (armagh or "armagh's" or belfast or "belfast's" or lisburn or "lisburn's" or londonderry or "londonderry's" or derry or "derry's" or newry or "newry's").ti,ab,in,ad.

38 or/31-37

39 (exp "arctic and antarctic"/ or exp oceanic regions/ or exp western hemisphere/ or exp africa/ or exp asia/ or exp "australia and new zealand"/) not (united kingdom/ or europe/)

40 38 not 39

41 8 and 12 and 23 and 40

42 8 and 12 and 30 and 40

43 limit 41 to yr="2014 -Current"

44 limit 42 to yr="2014 -Current"

Appendix C. Quality appraisal

Quality Appraisal ratings for qualitative studies using the NICE Quality Appraisal Checklist Appendix H (Spencer et al, 2003; Public Health Resource Unit, 2006; North Thames Research Appraisal Group, 1998)

| Quality Appraisal Criteria | Maternity action, 2018 | Feldman, 2020 | Gardner, 2021 | Healthwatch Hackney, 2020 | Murphy et aly, 2020 | Nellums et al, 2020 | Doctors of the World, 2020 | Shortall, 2015 | Patients not passports, 2020 |
| --- | --- | --- | --- | --- | --- | --- | --- | --- | --- |
| 1. Is a qualitative approach appropriate? | Appropriate | Appropriate | Appropriate | Appropriate | Appropriate | Appropriate | Appropriate | Appropriate | Appropriate |
| 2. Is the study clear in what it seeks to do | Clear | Clear | Mixed | Clear | Clear | Clear | Clear | Clear | Clear |
| 3. How defensible/rigorous is the research design/ methodology? | Indefensible | Indefensible | Unclear | Unclear | Defensible | Defensible | Defensible | Defensible | Unclear |
| 4. How well was the data collection carried out? | Appropriately | Appropriately | Not sure/inadequately reported | Not sure/inadequately reported | Appropriately | Appropriately | Appropriately | Appropriately | Not sure/inadequately reported |
| 5. Is the role of the researcher clearly described? | Unclear | Clear | Unclear | Unclear | Clearly described | Clearly escribe | Unclear | Not described | Not described |
| 6. Is the context clearly described? | Clear | Clear | Clear | Clear | Clear | Clear | Clear | Clear | Clear |
| 7. Were the methods reliable? | Not sure | Not  sure | Not sure | Not sure | Reliable | Reliable | Unreliable | Not sure | Not sure |
| 8. Is the data analysis sufficiently rigorous? | Not sure/not reported | Not sure/not reported | Not sure/not reported | Not sure/not reported | Rigorous | Rigorous | Not rigorous | Not rigorous | Not rigorous |
| 9. Is the data 'rich'? | Rich | Rich | Poor | Rich | Rich | Rich | Rich | Rich | Rich |
| 10. Is the analysis reliable | Unreliable | Unreliable | Not sure/not reported | Not sure/not reported | Not sure/not reported | Reliable | Not sure/not reported | Reliable | Not sure/not reported |
| 11. Are the findings convincing? | Convincing | Convincing | Convincing | Convincing | Convincing | Convincing | Convincing | Convincing | Convincing |
| 12. Are the findings relevant to the aims of the study? | Relevant | Relevant | Relevant | Relevant | Relevant | Relevant | Relevant | Relevant | Relevant |
| 13. Is there adequate discussion of any limitations encountered? | Inadequate | Inadequate | Inadequate | Inadequate | Adequate | Adequate | Adequate | Adequate | Inadequate |
| 14. How clear and coherent is the reporting of ethics | Appropriate | Approrpiate | Not sure/not reported | Not sure/not reported | Not sure/not reported | Appropriate | Not sure/not reported | Not sure/not reported | Not sure/not reported |
| 15. Overall rating | Low | Low | Low | Low | Medium | Medium | Low | Medium | Low |

Quality Appraisal ratings for quantitative studies using the NICE Quality Appraisal Checklist Appendix G (Jackson et al, 2006)

| Quality Appraisal Criteria | Murphy et al, 2020 | Potter et al, 2020 | Shortall, 2015 | Patients Not Passports, 2020 |
| --- | --- | --- | --- | --- |
| 1.1 Is the source population or source area well described? | ++ | ++ | ++ | ++ |
| 1.2 Is the eligible population or area representative of the source population or area? | + | ++ | + | ++ |
| 1.3 Do the selected participants or areas represent the eligible population or area? | + | + | + | + |
| 2.1 Selection of exposure (and comparison) group. How was selection bias minimised? | NA | ++ | NR | NR |
| 2.2 Was the selection of explanatory variables based on a sound theoretical basis? | NA | ++ | NA | NA |
| 2.3 Was the contamination acceptably low? | NA | NR | NA | NA |
| 2.4 How well were likely confounding factors identified and controlled? | NR | + | NR | NR |
| 2.5 Is the setting applicable to the UK? | ++ | ++ | ++ | ++ |
| 3.1 Were the outcome measures and procedures reliable? | + | + | + | + |
| 3.2 Were the outcome measurements complete? | + | ++ | + | + |
| 3.3 Were all the important outcomes assessed? | ++ | + | ++ | ++ |
| 3.4 Was there a similar follow-up time in exposure and comparison groups? | NA | NA | NA | NA |
| 3.5 Was follow-up time meaningful? | NA | NA | NA | NA |
| 4.1 Was the study sufficiently powered to detect an intervention effect (if one exists)? | NA | ++ | NA | NA |
| 4.2 Were multiple explanatory variables considered in the analyses? | NR | NR | NR | NR |
| 4.3 Were the analytical methods appropriate? | ++ | ++ | + | - |
| .6 Was the precision of association given or calculable? Is association meaningful? | NA | ++ | NA | NA |
| 5.1 Are the study results internally valid (i.e. unbiased)? | + | + | - | - |
| 5.2 Are the findings generalisable to the source population (i.e. externally valid)? | + | ++ | + | + |
| Overall rating | Low | Medium | Low | Low |

Appendix D – Prisma Checklist

*From: Page MJ, McKenzie JE, Bossuyt PM, Boutron I, Hoffmann TC, Mulrow CD, et al. The PRISMA 2020 statement: an updated guideline for reporting systematic reviews. BMJ 2021;372:n71. doi:*

*10.1136/bmj.n71*

| Section and Topic | Item number | Checklist Item | Page where item is reported |
| --- | --- | --- | --- |
| Title | | | |
| Title | 1 | Identify the report as a systematic review | 1 |
| Abstract | | | |
| Abstract | 2 | See the PRISMA 2020 for Abstracts checklist | 2 |
| Introduction | | | |
| Rationale | 3 | Describe the rationale for the review in the context of existing knowledge | 2-3 |
| Objectives | 4 | Provide an explicit statement of the objective (s) or question(s) the review addresses | 2-3 |
| Methods | | | |
| Eligibility criteria | 5 | Specify the inclusion and exclusion criteria for the review and how studies were grouped for the syntheses. | 3-4 |
| Information sources | 6 | Specify all databases, registers, websites, organisations, reference lists and other sources searched or consulted to identify studies. Specify the  date when each source was last searched or consulted. | 3 |
| Search strategy | 7 | Present the full search strategies for all databases, registers and websites including any filters and limits used | 15-16 |
| Selection process | 8 | Specify the methods used to decide whether a study met the inclusion criteria of the review, including how many reviewers screened each record  and each report retrieved, whether they worked independently, and if applicable, details of automation tools used in the process | 3 |
| Data collection process | 9 | Specify the methods used to collect data from reports, including how many reviewers collected data from each report, whether they worked  independently, any processes for obtaining or confirming data from study investigators, and if applicable, details of automation tools used in the  process. | 4 |
| Data items | 10a | List and define all outcomes for which data were sought. Specify whether all results that were compatible with each outcome domain in each  study were sought (e.g. for all measures, time points, analyses), and if not, the methods used to decide which results to collect | 3 |
|  | 10b | List and define all other variables for which data were sought (e.g. participant and intervention characteristics, funding sources). Describe any  assumptions made about any missing or unclear information. | 3 |
| Study risk of bias assessment | 11 | Specify the methods used to assess risk of bias in the included studies, including details of the tool(s) used, how many reviewers assessed each  study and whether they worked independently, and if applicable, details of automation tools used in the process | 4 |
| Effect measures | 12 | Specify for each outcome the effect measure(s) (e.g. risk ratio, mean difference) used in the synthesis or presentation of results | 6 |
| Synthesis methods | 13a | Describe the processes used to decide which studies were eligible for each synthesis (e.g. tabulating the study intervention characteristics and  comparing against the planned groups for each synthesis (item #5)). | 4 |
|  | 13b | Describe any methods required to prepare the data for presentation or synthesis, such as handling of missing summary statistics, or data  conversions. | 4 |
|  | 13c | Describe any methods used to tabulate or visually display results of individual studies and syntheses | 4 |
|  | 13d | Describe any methods used to synthesize results and provide a rationale for the choice(s). If meta-analysis was performed, describe the  model(s), method(s) to identify the presence and extent of statistical heterogeneity, and software package(s) used. | 4 |
|  | 13e | Describe any methods used to explore possible causes of heterogeneity among study results (e.g. subgroup analysis, meta-regression). | N/A |
|  | 13f | Describe any sensitivity analyses conducted to assess robustness of the synthesized results | N/A |
| Reporting bias assessment | 14 | Describe any methods used to assess risk of bias due to missing results in a synthesis (arising from reporting biases). | N/A |
| Certainty assessment | 15 | Describe any methods used to assess certainty (or confidence) in the body of evidence for an outcome. | N/A |
| Results | | | |
| Study selection | 16a | Describe the results of the search and selection process, from the number of records identified in the search to the number of studies included in  the review, ideally using a flow diagram. | 5 |
|  | 16b | Cite studies that might appear to meet the inclusion criteria, but which were excluded, and explain why they were excluded | 5 |
| Study characteristics | 17 | Cite each included study and present its characteristics. | 8-10 |
| Risk of bias in studies | 18 | Present assessments of risk of bias for each included study | 17-20 |
| Results of individual studies | 19 | For all outcomes, present, for each study: (a) summary statistics for each group (where appropriate) and (b) an effect estimate and its precision  (e.g. confidence/credible interval), ideally using structured tables or plots | 8-10 |
| Results of synthesis | 20a | For each synthesis, briefly summarise the characteristics and risk of bias among contributing studies | 6 + 12 |
|  | 20b | Present results of all statistical syntheses conducted. If meta-analysis was done, present for each the summary estimate and its precision (e.g.  confidence/credible interval) and measures of statistical heterogeneity. If comparing groups, describe the direction of the effect. | N/A |
|  | 20c | Present results of all investigations of possible causes of heterogeneity among study results. | N/A |
|  | 20d | Present results of all sensitivity analyses conducted to assess the robustness of the synthesized results | N/A |
| Reporting biases | 21 | Present assessments of risk of bias due to missing results (arising from reporting biases) for each synthesis assessed | 16-19 |
| Certainty of evidence | 22 | Present assessments of certainty (or confidence) in the body of evidence for each outcome assessed. | N/A |
| Discussion | | | |
| Discussion | 23a | Provide a general interpretation of the results in the context of other evidence. | 12 |
|  | 23b | Discuss any limitations of the evidence included in the review | 13-14 |
|  | 23c | Discuss any limitations of the review processes used | 13-14 |
|  | 23d | Discuss implications of the results for practice, policy, and future research | 13 |
| Other information | | | |
| Registration and protocol | 24a | Provide registration information for the review, including register name and registration number, or state that the review was not registered. | 3 |
|  | 24b | Indicate where the review protocol can be accessed, or state that a protocol was not prepared. | 3 |
|  | 24c | Describe and explain any amendments to information provided at registration or in the protocol | 13 |
| Support | 25 | Describe sources of financial or non-financial support for the review, and the role of the funders or sponsors in the review | 14 |
| Competing interests | 26 | Declare any competing interests of review authors | 14 |
| Availability of data, code and other materials | 27 | Report which of the following are publicly available and where they can be found: template data collection forms; data extracted from included studies; data used for all analyses; analytic code; any other materials used in the review. |  |
